# Geographic Accessibility of Deceased Organ Donor Care Units

**DOI:** 10.1001/jamanetworkopen.2026.1703

**Published:** 2026-03-13

**Authors:** Vishnu S. Potluri, Vicky Tam, Elizabeth M. Sonnenberg, Richard D. Hasz, Joel T. Adler, Douglas J. Wiebe, Peter P. Reese, Emily A. Vail

**Affiliations:** 1 Renal-Electrolyte and Hypertension Division, Department of Medicine, Hospital of the University of Pennsylvania, Philadelphia; 2 Solid Organ Transplantation Epidemiology, Ethics, and Policy Research Group, Philadelphia, Pennsylvania; 3 Leonard Davis Institute of Health Economics, University of Pennsylvania, Philadelphia; 4 Data Science and Biostatistics Unit, Children’s Hospital of Philadelphia, Philadelphia, Pennsylvania; 5 Department of Surgery, Hospital of the University of Pennsylvania, Philadelphia; 6 Gift of Life Donor Program, Philadelphia, Pennsylvania; 7 Division of Transplantation, Department of Surgery and Perioperative Care, Dell Medical School at The University of Texas at Austin; 8 Department of Epidemiology, University of Michigan, Ann Arbor; 9 Vanderbilt Center for Transplant Science, Vanderbilt University, Nashville, Tennessee; 10 Department of Anesthesiology and Critical Care, Hospital of the University of Pennsylvania, Philadelphia

## Abstract

**Importance:**

Transfers of deceased organ donors from acute care hospitals to specialized donor care units (DCUs) offer operational and outcome advantages; however, current access to DCUs is limited and geographically uneven. Expanding access to DCUs may improve donation system efficiency.

**Objective:**

To evaluate the geographic distribution of operating DCUs relative to acute care hospitals and explore how to most efficiently operationalize recommendations that a DCU operate in every donation region.

**Design, Setting, and Participants:**

This retrospective cohort study analyzed deceased organ donor and hospital data captured in the Organ Procurement and Transplantation Network and American Hospital Association survey databases from January 1, 2018, to December 31, 2023. Acute-care hospitals and DCUs operating in the continental US and adult (aged ≥18 years) organ donors with brain death managed in acute care hospitals located in 2203 zip codes were included. The data analysis was performed between October 1, 2024, and December 1, 2025.

**Exposures:**

Geographic location of organ donor hospitals.

**Main Outcomes and Measures:**

The main outcome was the optimal number of DCUs required to enable transportation of all cohort donors from acute care hospitals to DCUs via ambulance (within a 180-minute drive). The number of additional DCUs needed to operationalize recommendations of a DCU in every donation region was quantified with and without consideration for donation service area boundaries using location-allocation modeling.

**Results:**

Between 2018 and 2023, 53 093 deceased donors met the inclusion criteria (mean [SD] age, 44.3 years [15.0]; 60.0% male). Among the cohort, 61.9% of donors were managed in acute care hospitals within driving distance of 34 operating DCUs. In the current system with distinct donation service area boundaries, an additional 38 DCUs were estimated to provide plausible access to 92.7% of donors. If donation service area boundaries were ignored, 22 new DCUs were estimated to provide a referral facility for a larger proportion of donors (96.5%).

**Conclusions and Relevance:**

This cohort study found that despite their reported advantages and consensus endorsement, heterogeneous adoption of DCUs has left a substantial proportion of deceased donors after brain death more than a 180-minute drive from a DCU. Given inefficiencies introduced by donation service area boundaries, opening additional DCUs in acute care hospitals and donor transport across these existing boundaries may be 2 potential approaches to improve system efficiency and donation outcomes.

## Key Points

**Question:**

Where should deceased organ donor care units (DCUs) be located to maximize hospital access and minimize transport time and distance from referring hospitals?

**Findings:**

In this cohort study of 53 093 organ donors with brain death in the continental US, 61.9% were from hospitals within a 180-minute drive of an operating DCU. Opening 38 additional hospital-based DCUs (while respecting service boundaries) was estimated to cover 92.7% of donors, whereas 22 additional DCUs (while ignoring boundaries) was estimated to cover 96.5%.

**Meaning:**

These findings uncovered an unmet need to improve donor hospital access to centralized donor management in DCUs and suggest that sharing of the existing health care infrastructure may be leveraged to address this gap.

## Teaser

This cohort study evaluates the geographic distribution of operating US donor care units within driving distance of acute care hospitals and estimates the additional number needed to maximize access and minimize transport time.

## Introduction

The US organ donation and transplant systems are undergoing rapid structural, operational, and allocation policy changes aimed at improving patients’ access to transplantable organs. Over the past 2 decades, to address well-documented challenges of clinical deceased organ donor management and recovery in acute care hospitals,^1^ some regional donation organizations have transported potential organ donors after brain death from hospitals to specialized clinical management and organ recovery facilities: donor care units (DCUs).^2^ A DCU offers logistical and some outcome advantages over traditional donor management in acute care hospitals, including better adherence to clinical management protocols; lower donor management costs; and, in some cases, more organs recovered for transplantation.^3,4,5^ Therefore, the National Academies of Sciences, Engineering, and Medicine recommend that all organ procurement organizations (OPOs), which are federal contractors responsible for organ donor identification, management, organ allocation, and recovery within specific regions (termed donation service areas), operate DCUs.^6^

Despite their advantages, national DCU availability and use vary for two reasons. First, OPOs open and operate these units at their discretion. Without Health Resources and Services Administration or Centers for Medicare & Medicaid Services (CMS) mandates, OPOs choose whether to open DCUs according to operational needs and resources (both financial and regional health system resources). Therefore, adoption is incomplete.^7,8^ Second, it is rare for donors to be transferred between donation service areas, even those with a nearby DCU.

Donation service areas are geographic areas designated by CMS, which include 1 or more donor hospitals served by a single OPO and at least 1 transplant program.^9^ Area boundaries are irregular and, in some cases, noncontiguous; they reflect historical service contracts between OPOs and individual donor hospitals.^10^ Although donation service areas were not developed for efficient organ allocation, they were initially used for this purpose.^11^ In the intervening decades, legal challenges and input from stakeholder groups have changed allocation policies for most solid organ types to prioritize minimization of geographic distance between donors and potential recipients (to reduce organ ischemic time while in transit) over adherence to area boundaries.^12,13^ Despite organ allocation rule changes and DCU adoption, donation service area boundaries have only changed in response to OPO mergers.^14^ These resulting DCU access gaps are a potential source of system inefficiency.^7^

Geographic information systems are spatial datasets and analysis methods that offer unique opportunities to understand associations among donor hospitals, DCUs, and donation service areas. Location-allocation modeling is one geographic information system application routinely used in health care to optimize population access to emergency medical services, such as trauma and stroke care.^15,16^ Location-allocation modeling may offer insights into optimal organization and distribution of clinical services required for donor management (eg, coronary catheterization laboratories) that are not available in all US hospitals but may be consolidated into DCUs.^17^ Using these methods, we sought to identify the optimal number and locations of DCUs required to transfer all potential donors after brain death from hospitals efficiently.

## Methods

This cohort study used data from the Organ Procurement and Transplantation Network (OPTN). The OPTN data system includes data on all donors, wait-listed candidates, and transplant recipients in the US, submitted by the members of the OPTN. The Health Resources and Services Administration, US Department of Health and Human Services, provides oversight to the activities of the OPTN contractor. The University of Pennsylvania Institutional Review Board approved the study and deemed it exempt from human participant research because it used an existing dataset that captured only decedents. The study adhered to the Strengthening the Reporting of Observational Studies in Epidemiology (STROBE) reporting guideline.

We assembled a cohort of deceased adult (aged ≥18 years) organ donors (as pediatric donors and donors after circulatory death are rarely transferred to DCUs) captured in the OPTN dataset between January 1, 2018, and December 31, 2023. We excluded donors missing hospital locations and those who underwent organ recovery outside the continental US. Race and ethnicity were recorded by organ transplant coordinators at the time of organ donor assessment. Race and ethnicity categories included American Indian or Alaska Native, Asian, Black, Hispanic, Native Hawaiian or Other Pacific Islander, White, and multiracial. The study assessed these characteristics because of known differences in rates of deceased organ donation among groups.^18^

Given the ongoing US expansion of hospital-based DCUs,^2,19^ we considered operating acute care hospitals as potential new DCU sites (hereafter referred to as candidate DCU sites). Hospital characteristics and locations were identified in the American Hospital Association (AHA) Annual Survey Database (2019-2022), which compiles information from the AHA Annual Survey of Hospitals (a voluntary survey distributed to all US hospitals), government sources, hospital accreditors, and other organizations.^20^ We excluded hospitals without key clinical services routinely used in deceased donor management and evaluation, including coronary catheterization, hemodialysis, and intensive care unit beds. The OPTN dataset defined hospitals with transplant programs; the AHA dataset defined all other characteristics. We also excluded pediatric specialty hospitals, which are unlikely to accept transfers of adult potential donors. Although hospitals with transplant programs have specific financial incentives to operate DCUs,^21^ the analysis focused on the possible expansion of DCU coverage to improve donors’ access to comprehensive management; therefore, we did not exclude hospitals without transplant programs. We excluded hospitals outside the continental US and hospitals more than a 30-minute drive from a Federal Aviation Administration–designated primary airport (a National Academies of Sciences, Engineering, and Medicine report recommendation)^6^ to ensure expedited air travel for recovering surgical teams and recovered organs. Finally, given our expectation that operating DCUs are unlikely to change locations, we excluded hospitals within a 60-minute drive of an existing DCU.

### Geospatial Modeling

After selecting organ donor and donor hospital cohorts, we mapped the distributions of cohort donors using hospital zip codes and locations of existing DCUs by street addresses. Existing DCUs were restricted to those operating in the continental US by the end of the study period.^2,7,19^ Next, assuming that most donors would be transported from hospitals to DCUs by ambulance, we estimated road network driving times between population-weighted centroids of each donor hospital zip code and existing and candidate DCU sites (assuming optimal weather and traffic conditions) using ArcGIS Pro, version 3.2 (Esri). Finally, we generated location-allocation models (using the maximize coverage and minimize facilities optimization method) to address 2 scenarios: donor transfers from hospitals to a DCU located within donation service area boundaries (model 1) and across boundaries (model 2) (Table 1). For both models, we made the following 3 assumptions: (1) ground transfers up to 180 minutes were clinically feasible; (2) if a DCU were available, all nearby donors would be transferred there; and (3) a minimum number of donor transfers would be necessary for a DCU transfer and management to be clinically and financially superior to hospitals. Thus, we excluded candidate DCUs with fewer than 200 nearby donors after brain death during the 6-year study period. Both models used an exponential decay function that assigned greater weights to nearby organ donor hospital zip codes. This approach minimizes the overall travel burden while systematically diminishing the influence of remote hospitals.^22^ Additional details are available in the eMethods in Supplement 1.

**Table 1.** Factors Used to Estimate the Placement of DCUs in the Current State and Proposed Systems

Analysis	Current state^a^	Model 1: donors are not transported across OPO boundaries	Model 2: donors may be transported across OPO boundaries
Factors used to determine DCU locations	Location and type (independent or hospital-based) selected by OPO	Acute care hospital; ≥1 adult ICU bed; coronary catheterization laboratory; capacity to deliver kidney replacement therapy; within a 30-min drive of an FAA-designated airport; and >60-min drive from an existing DCU	Acute care hospital; ≥1 adult ICU bed; coronary catheterization laboratory; capacity to deliver kidney replacement therapy; within a 30-min drive of an FAA-designated airport; and >60-min drive from an existing DCU
Donor transfer across OPO boundaries	Not available^b^	Not allowed	Allowed
Minimum No. of DCUs per DSA region	0-2	1^c^	NA
Maximum No. of DCUs per DSA region	No limit	No limit	NA
Maximum road travel time between hospitals and DCU, min	Established by individual OPOs	180	180
Minimum donor volume during study period (2018-2023)	Established by individual OPOs	>200	>200

Abbreviations: DCU, donor care unit; DSA, donation service area; FAA, Federal Aviation Administration; ICU, intensive care unit; NA, not applicable; OPO, organ procurement organization.

^a^ As of December 31, 2023; included 12 independent and 22 hospital-based existing DCUs.

^b^ Anecdotally, donor transfer across OPO boundaries is rare.

^c^ Except for DSAs not satisfying a minimum volume criterion of 200 donors potentially transferred during the study period.

### Statistical Analyses

The data analysis was performed between October 1, 2024, and December 1, 2025. Each model identified optimal locations of proposed DCUs from candidate DCU sites. After model building, we mapped the locations of proposed DCUs and compared the characteristics of the current network of existing DCUs and referring hospitals with each model (comprising existing and proposed DCUs). The primary outcome was the optimal number and locations of DCUs required to satisfy the assumptions of each geospatial model. Secondary outcomes were broadly organized into 3 categories: (1) donor transportation efficiency, (2) geographic coverage, and (3) spatial distribution. We evaluated donor transportation efficiency based on road transport times and distances between donor hospitals and the group of existing and proposed DCU locations, weighted by the number of cohort donors in each hospital (eMethods in Supplement 1). We also calculated driving distances between DCUs and the nearest hospital with a transplant program. We defined geographic coverage according to the proportion of cohort donors and cohort hospitals within prespecified road network drive times of at least 1 DCU.

To evaluate spatial distribution, we calculated the average nearest neighbor index for each model to assess whether DCU locations showed statistically significant clustering or dispersion.^23,24,25^ The average nearest neighbor index measures the distances between each DCU and its closest neighbor DCU and compares the average of those distances against a hypothetical random distribution. A value of less than 1 indicates a network that is more clustered than a random distribution, while a value greater than 1 indicates that the network is more dispersed than a random network.

As reasonable drive times (between donor hospitals and DCUs and between DCUs and airports) remain undefined, we performed 3 sensitivity analyses. First, we estimated the number of candidate acute care hospitals near airports in 15-minute drive time increments up to 60 minutes (which we considered as a maximum reasonable time for this component of organ transport). Next, modifying the model that ignored donation service area boundaries, we increased the maximum drive time from a DCU to an airport to 45 minutes (model 3) and increased the donor transport time threshold to 240 minutes (model 4). Missing data were minimal and not imputed. Data were analyzed using Stata, version 18.0 (StataCorp LLC).

## Results

### Cohort and Current Donation System Overview

Between 2018 and 2023, 53 093 deceased adult organ donors with brain death (mean [SD] age, 44.3 [15.0] years; 40.0% female and 60.0% male; 2.9% of Asian, 17.9% of Black, 16.4% of Hispanic, 61.4% of White, and 1.4% of other [American Indian or Alaska Native, Native Hawaiian or Pacific Islander, or multiracial] race and ethnicity) were managed in acute care hospitals located in 2203 US zip codes (eFigure 1 in Supplement 1). At the end of the study period, 34 DCUs (22 hospital-based and 12 independent) were operating in 32 donation service areas, and 61.9% of the donors were located within a 180-minute drive of an operating DCU in the same donation region.

**Figure 1.**
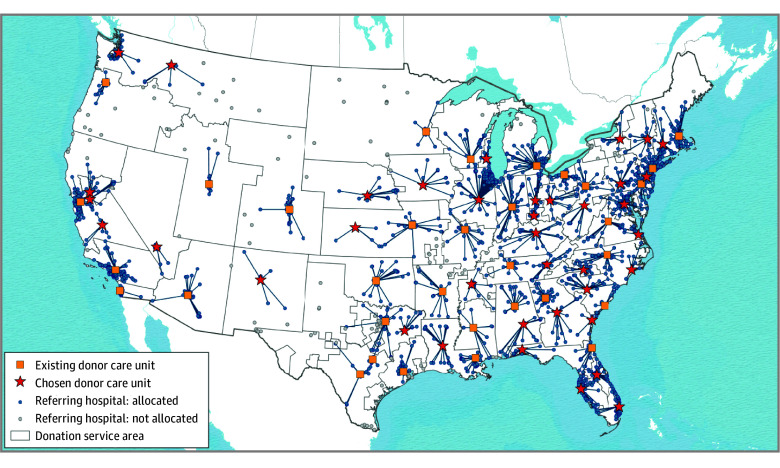
Map Showing Proposed Locations of New Hospital-Based Donor Care Units to Manage Cohort Donors, Restricting Transport of Donors Within Donation Service Area Boundaries (Model 1) Donation service area boundaries were defined by the Scientific Registry of Transplant Recipients.

**Figure 2.**
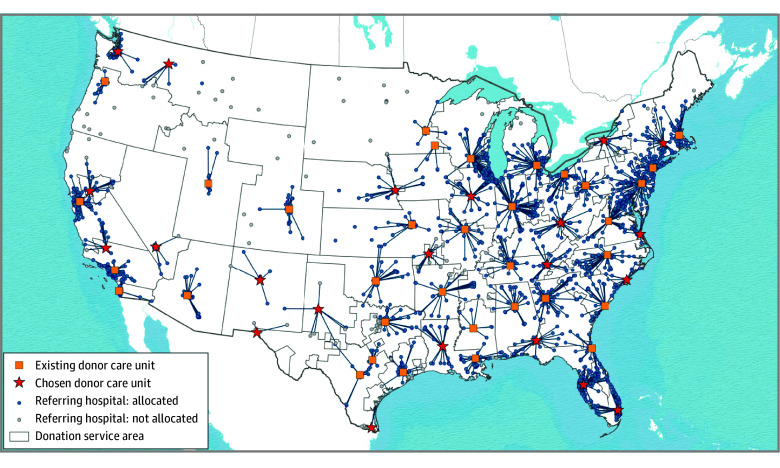
Map Showing Proposed Locations of New Hospital-Based Donor Care Units to Manage Cohort Donors, Allowing Transport of Donors Across Donation Service Area Boundaries (Model 2) Donation service area boundaries were defined by the Scientific Registry of Transplant Recipients.

A total of 1199 acute care hospitals in the AHA database with clinical services sufficient for managing and evaluating adult organ donors with brain death met candidate DCU site inclusion criteria. After geographic exclusions, 438 candidate hospitals remained (eFigure 2 in Supplement 2). Figure 1 and Figure 2 include maps of existing DCUs, candidate hospitals, and proposed DCUs for each model.

### Model 1: Expanding DCU Locations Within Existing Donation Service Area Boundaries

In the model accounting for donation service area boundaries, 38 additional hospital-based DCU sites were proposed for a total of 72 DCUs across 54 donation regions (Table 2). Our model did not identify a DCU location in 1 donation service area (located in the northeastern US), in which the number of donors in nearby hospitals did not satisfy the a priori donor volume criterion.

**Table 2.** Comparison of Donor Transportation Efficiency and Geographic Coverage Among Current State and Location-Allocation Models

Outcome	Current state^a^	Model 1: respecting OPO boundaries	Model 2: ignoring OPO boundaries
**Primary outcome**			
No. of DCUs			
All	34	72	56
Existing	34	34	34
Proposed	NA	38	22
**Secondary outcomes**			
Donors within 180 min of a DCU, No. (%)^b^	32 862 (61.9)	49 222 (92.7)	51 213 (96.5)
Donor hospital zip codes within 180 min of a DCU, No. (%)	1305 (59.2)	2011 (91.3)	2095 (95.1)
Donors within 180 min of each (existing and proposed) DCU, median (IQR)	812 (566-1262)	609 (366-837)	762 (430-1138)
Donors covered by each projected DCU, median (IQR)	NA	412 (288-750)	475 (333-762)
Donor hospital zip codes within 180 min of a DCU, median (IQR)	33.5 (21.3-44.5)	22.0 (16.0-34.5)	30.0 (18.8-46.3)
Drive time between donor hospitals and DCUs, median (IQR), min^c^	41.7 (18.4-96.1)	37.9 (16.0-79.0)	50.5 (19.9-105.9)
Driving distance between donor hospitals and DCUs, median (IQR), miles^c^	34.3 (9.7-88.8)	29.1 (8.6-72.2)	40.8 (11.3-105.2)

Abbreviations: DCU, donor care unit; NA, not applicable; OPO, organ procurement organization.

^a^ As of December 31, 2023; included 12 independent and 22 hospital-based existing DCUs. For distance calculations, when a donor was located within the 180-minute boundary of 2 DCUs in the same donor service area, they were attributed to the nearest DCU.

^b^ Donor volume was calculated assuming that all donors within a 180-minute drive were transferred over the study period (2018-2023).

^c^ Weighted by number of donors in each hospital.

Across the continental US, complete adoption of this model was estimated to increase the number of cohort donors within a 180-minute drive of an existing or new DCU from 32 862 (61.9%) to 49 222 (92.7%). Model expansion was projected to increase the number of zip codes with acute care hospitals near a DCU from 1305 (59.2%) in the current state to 2011 (91.3%). The median number of donors potentially managed at each DCU was estimated to decrease from 812 (IQR, 566-1262) to 609 (IQR, 366-837). Median transport times between hospitals and DCUs (weighted for the proportion of cohort donors in each referring hospital) remained similar nationally (from 41.7 [IQR, 18.4-96.1] minutes in the current state to 37.9 [IQR, 16.X-79.X] minutes after model adoption). The median distance from DCUs to the nearest transplant center was 7.1 miles (IQR, 0.4-48.4 miles) in model 1.

### Model 2: Allowing Donor Transfers Across Donation Service Area Boundaries

In the second model, 22 new DCU sites were proposed (56 total). In this model, the number and proportion of cohort donors covered by a DCU increased from 32 862 (61.9%) to 51 213 (96.5%). This model also projected an increase in the proportion of US hospital zip codes within a 180-minute drive of a DCU from 1305 (59.2%) to 2095 (95.1%). The median number of donors within 180 minutes of existing and proposed DCUs in the model was 762 (IQR, 430-1138). In this model, the donor-weighted median transport time between hospitals and DCUs increased nationally from 41.7 minutes (IQR, 18.4-96.1 minutes) to 50.5 minutes (IQR, 19.9-105.9 minutes). The median distance from DCUs to the nearest transplant center was 5.6 miles (IQR, 0.4-49.7 miles) in model 2.

### Model Comparisons

**Figure 3.**
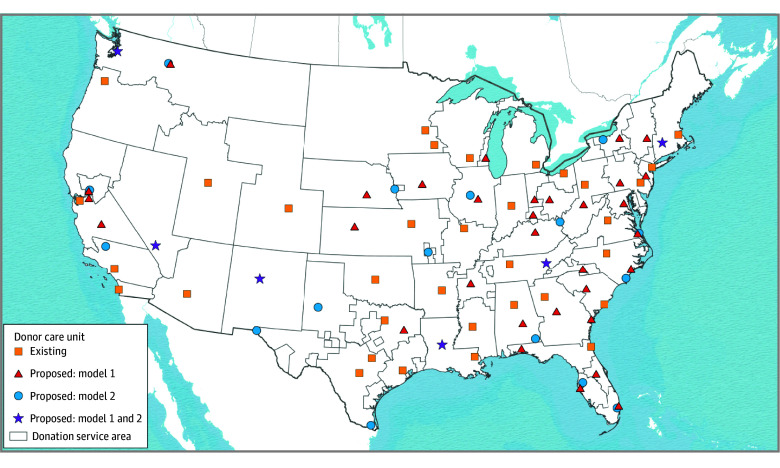
Map Showing the Comparison of Existing Proposed Donor Care Unit (DCU) Locations With and Without Current Donation Service Area Boundaries (Models 1 and 2) Model 1 preserves existing organ procurement organization region (donation service area) boundaries, as defined by the Scientific Registry of Transplant Recipients. Model 2 disregards existing donation service area boundaries. Additional model assumptions are described in Table 1 and the eMethods in Supplement 1. Interactive maps illustrating candidate hospitals, existing DCUs, and proposed DCUs for both models are also available.^26^

Without respecting DSA boundaries, model 2 proposed 16 (42.1%) fewer new DCUs compared with model 1. Figure 3 compares the numbers and locations of existing and proposed DCUs between models (72 in model 1 vs 56 in model 2). When donation area boundaries were disregarded, slightly more cohort donors (51 213 [96.5%] vs 49 222 [92.7%]) had geographic coverage overall. The median number of cohort donors managed in each DCU was also higher when boundaries were ignored (762 [IQR, 430-1138] vs 609 [IQR, 366-837] in model 1).

Model 2, which allowed donor transfers across donation service area boundaries, captured more donor hospital zip codes covered by a nearby DCU compared with model 1 (2095 vs 2011, respectively). In terms of transportation efficiency, median donor transport time between donor hospitals and new and existing DCUs (weighted for the number of donors in each hospital) was 37.9 minutes (IQR, 16.0-79.0 minutes) in model 1 and 50.5 minutes (IQR, 19.9-105.9 minutes) in model 2. The average nearest neighbor index in both models was greater than 1, indicating statistically significant dispersion of existing and new DCUs (1.21 in model 1 and 1.34 in model 2).

Results from sensitivity analyses are presented in eTables 1 and 2 in Supplement 1. Increasing the drive time threshold from a DCU to an airport in 15-minute increments progressively increased the number of candidate hospitals. Increasing the maximum drive times to an airport from 30 to 45 minutes (model 3) did not yield meaningful changes in primary or secondary outcomes. However, increasing the maximum drive time threshold from donor hospitals to DCUs to 240 minutes (model 4) reduced the number of proposed DCUs to 17, while increasing the median weighted drive time between donor hospitals and DCUs to 59.4 minutes (IQR, 21.8-112.9 minutes), vs 50.5 minutes (IQR, 19.9-105.9 minutes) in model 2, when a 180-minute threshold was used.

## Discussion

This retrospective cohort study that analyzed US national deceased organ donor and hospital data estimated that only 61.9% of adult donors with brain death were managed in hospitals within a 180-minute drive time of an operating DCU. To further improve geographic coverage and transport efficiency to these units, we examined 2 complementary potential solutions: (1) opening new DCUs in acute care hospitals with sufficient donor management resources and (2) transporting deceased donors across donation service area boundaries to nearby DCUs. Using established geospatial modeling approaches, we estimated that opening 38 additional DCUs inside acute care hospitals would increase the proportion of covered donors to more than 90%. If donors were regularly transferred across DSA boundaries, only 22 new DCUs were estimated to achieve a similar degree of coverage.

This study represents a novel application of a widely used geospatial epidemiology approach to optimize donor coverage and enhance access to high-quality organ donor management for donor hospitals. It builds on past work showing variation in the distribution of deceased donors in hospitals across the US^27,28^ and growing evidence of superior operational outcomes for donors transferred to DCUs vs hospitals (and in some cases, recipients of organs recovered there).^3,4,5,8,29,30,31^ Donor care units may overcome logistical challenges common in acute care hospitals, such as variable donor management experience^1^ and delayed clinical testing (eg, tissue biopsies) required for timely and efficient organ allocation and recovery. Because potential donors in DCUs with dedicated resources may be less likely to compete with living patients for limited hospital resources, such as intensive care unit beds, attention from busy clinicians, and operating rooms, they may also mitigate regional hospital strain. Centralized donor care also allows OPOs to directly control more donor care costs by eliminating unnecessary equipment overhead and independently negotiating with contractors and suppliers.^5^

The study also extends previous research by Vail et al^7^ by showing that OPO boundaries introduce geographic inefficiency by limiting transfers of some potential donors to nearby DCUs. That study estimated that more than 3000 deceased adult donors after brain death (9.5% of cohort donors from 2017 to 2021) were within a 120-minute drive of a DCU operating in a nearby donation service area. The authors suggested that neighboring OPOs may choose to transfer donors to nearby service areas with available DCUs by executing contracts for specific donor management services, which would have the additional benefit of preserving long-standing relationships between area donor hospitals and OPOs. However, ongoing efforts to reorganize the donation and transplant system^32^ may offer different opportunities to revisit donation service area boundaries and their roles.

Our analyses also illustrate that the paradigm of 1 DCU per OPO proposed by the National Academies report^6^ may be insufficient in some regions and redundant in others. This finding is consistent with current practice, as some OPOs already operate more than 1 DCU, and further illustrates potential DCU sharing opportunities, particularly between OPOs with lower donor volumes or those whose boundaries extend through densely populated areas. An alternative perspective is that expanding the number of DCUs beyond an efficiency-defined minimum may mitigate travel and logistical burdens (eg, funeral arrangements) for donor families, although rigorous qualitative studies of donor family experiences and needs are required.

### Limitations

Our study has several limitations. First, it is not yet known which DCU features (eg, volume, staffing, affiliated hospital resources) may influence donation and transplantation outcomes. Single-center reports of DCU-facilitated organ interventions, such as dialysis and prone positioning,^33,34^ suggest that expanded DCU use may ultimately increase the number and quality of organs accepted for transplant, but evidence remains nascent. Second, our models used historical donor and hospital data, which cannot account for changes in donor distributions or donation practices over time or in the future. Other temporal changes, such as the introduction of CMS OPO performance measures^35^ and new organ allocation rules,^13^ may have led to changes in OPO and transplant program practices, including decisions to open or operate a DCU. More specifically, the study did not account for the rapid growth of donation after circulatory death in the US.^36^ While donation after circulatory death management capacity is expanding, additional resources required for transfer and comprehensive care of these patients have delayed their routine transfer to most operating DCUs.^2,19,37^ Third, while we recognize that most hospital-based DCUs operate in hospitals with transplant programs, this analysis focused on expanding geographic coverage; thus, we did not exclude US hospitals without transplant programs (the majority).^2,19^ Fourth, while donor transfer rates vary among OPOs,^7^ we assumed that donor transport time and distance are the only factors associated with use of existing DCUs, potentially overestimating the number of donors transferred to proposed DCUs. While DCU expansion may lead to more donor transfers, this assumption could not be tested using the available data or account for current barriers to transfers of individual donors (eg, clinical instability, coroner investigations) or hospital decisions to prevent transfers (in most cases, associated with perceived financial penalties for donors moved from hospitals with transplant programs).^21^ Finally, we could not account for unmeasured and dynamic factors, such as hospital case mix, staffing, and strain, that may preclude the opening of DCUs in hospitals selected by the model or influence the likelihood of donor transfer there. Similarly, we could not account for financial or operational factors, such as startup and operating costs, expected DCU outcomes, or capacity to accept transfers of potential deceased donors, that OPO leaders may consider when determining whether to open or expand DCUs. Despite these limitations, our model was designed to ensure that proposed new DCU locations would be in or near hospitals with the highest historical density of donors and with sufficient resources to continuously support donor assessment, management, and transportation of recovery teams and organs.

## Conclusions

By applying a widely used geographic modeling approach, this cohort study of organ donors after brain death and acute care hospitals in the US uncovered opportunities to expand centralized donor management coverage and efficiency of the deceased donation system by opening new DCUs in acute care hospitals and, in some cases, transporting potential donors between donation service areas. Ensuring that centralized donor management meets or exceeds its promised value will require sustained investment in infrastructure and policy changes to facilitate the dissemination of best practices, resource sharing, and cooperation among professional system stakeholders.

## Notes

Supplement 1.
eMethods.
**eFigure 1.** Distribution of Cohort Organ Donors Among US Acute Care Hospitals **eFigure 2.** Candidate Donor Care Unit Selection Diagram **eTable 1.** Number of Candidate DCUs According to Maximum Driving Time From a DCU to an Airport **eTable 2.** Comparison of Transportation Efficiency and Geographic Coverage Following an Increase in Driving Time Boundaries
eReferences.

Supplement 2.
Data Sharing Statement
