# Supplementary material for: Geographic Accessibility of Deceased Organ Donor Care Units
Source: JAMA Netw Open. 2026 Mar 13;9(3):e261703. doi: 10.1001/jamanetworkopen.2026.1703 (PMC12988448; doi:10.1001/jamanetworkopen.2026.1703)
Supplement: Supplement 1. — eMethods. eFigure 1. Distribution of Cohort Organ Donors Among US Acute Care Hospitals eFigure 2. Candidate Donor Care Unit Selection Diagram eTable 1. Number of Candidate DCUs According to Maximum Driving Time From a DCU to an Airport eTable 2. Comparison of Transportation Efficiency and Geographic Coverage Following an Increase in Driving Time Boundaries eReferences. [file jamanetwopen-e261703-s001.pdf]

Supplemental Online Content

Potluri VS, Tam V, Sonnenberg EM, et al. Geographic accessibility of deceased organ donor care units. *JAMA Netw Open*. 2026;9(3):e261703. doi:10.1001/jamanetworkopen.2026.1703

eMethods.

eFigure 1. Distribution of Cohort Organ Donors Among US Acute Care Hospitals

eFigure 2. Candidate Donor Care Unit Selection Diagram

eTable 1. Number of Candidate DCUs According to Maximum Driving Time From a DCU to an Airport

eTable 2. Comparison of Transportation Efficiency and Geographic Coverage Following an Increase in Driving Time Boundaries

eReferences

This supplemental material has been provided by the authors to give readers additional information about their work.

© 2026 Potluri VS et al. *JAMA Network Open*.

eMethods.

Model assumptions

All models accounted for US DCUs operating during the study period by excluding candidate DCU hospitals within a 60-minute drive of an existing facility. Based on past work that did not identify the distance between a donor hospital and DCU as a factor associated with the likelihood of transfer of donors after brain death,[1] we assumed that these donors could be transferred up to 180 minutes (by ambulance) from donor hospitals to candidate DCUs. Furthermore, given that most DCUs cover opening and operational expenses by accepting larger volumes of potential donors, we excluded candidate DCU sites when fewer than 200 cohort donors were near those sites. Additionally, the ‘Model 2’ assumed that potential donor families would not consider OPO boundaries in decisions to transfer loved ones to a DCU and allowed transfers of donors across OPO boundaries. Finally, our models assumed a 100% transfer rate to a DCU, as our objective was to identify the optimal number of DCUs required to achieve maximal coverage. While DCU adoption across the nation continues to increase, we acknowledge that prior work by Marklin et al. and our group, has demonstrated substantial variation in transfer rates to a DCU.[1,2]

Donor transportation efficiency measures

We estimated national donor-weighted distributions of driving time (in minutes) and distance (in miles) from donor hospitals to their allocated donor care unit (DCU). Each observation represented a donor hospital–DCU pair with associated driving time and distance. To account for differential donor volume across hospitals, ewe weighted ach hospital by the number of donors. Weighted quantiles were calculated by sorting observations by the outcome (e.g., driving time or distance), then calculating the cumulative sum of donor weights, and identifying the smallest outcome value at which the cumulative donor weight reached or

© 2026 Potluri VS et al. *JAMA Network Open*.

exceeded the desired percentile of the total donor weight. Using this approach, we estimated medians and interquartile ranges for both driving time and distance.

© 2026 Potluri VS et al. *JAMA Network Open*.

eFigure 1. Distribution of Cohort Organ Donors Among US Acute Care Hospitals

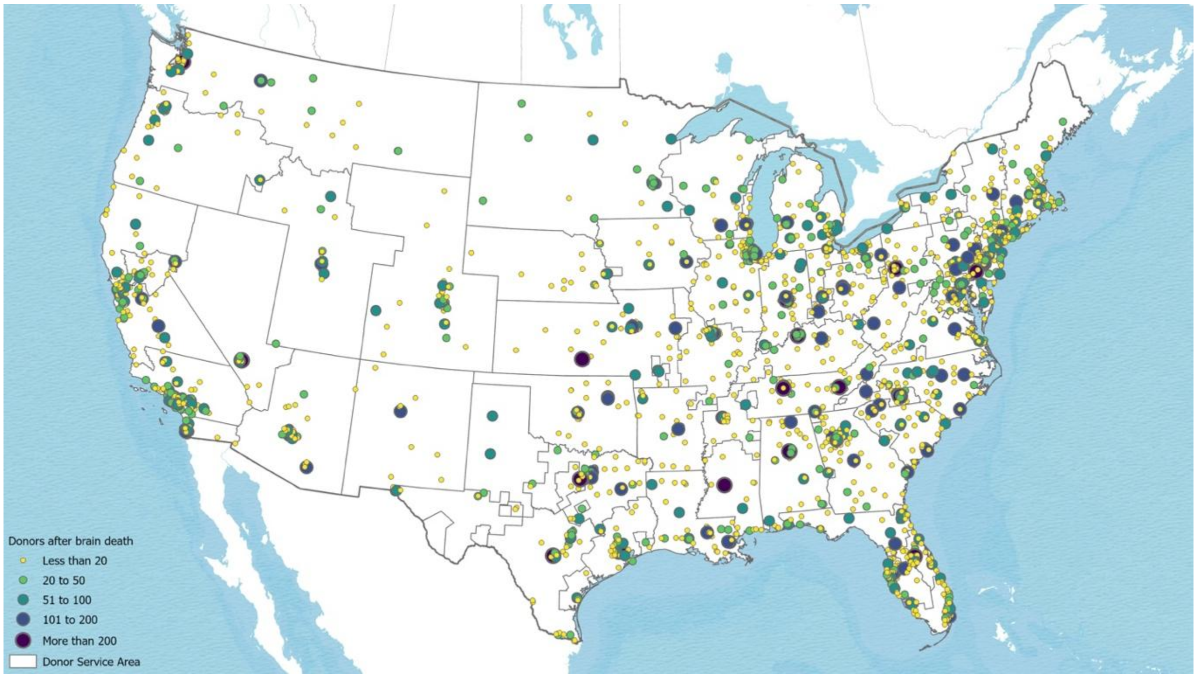

*Hospitals outside of the continental US were excluded from all analyses. Does not distinguish between donors managed exclusively in acute-care hospitals and those transferred to DCUs for clinical management and/or organ recovery.

© 2026 Potluri VS et al. *JAMA Network Open*.

eFigure 2. Candidate Donor Care Unit Selection Diagram

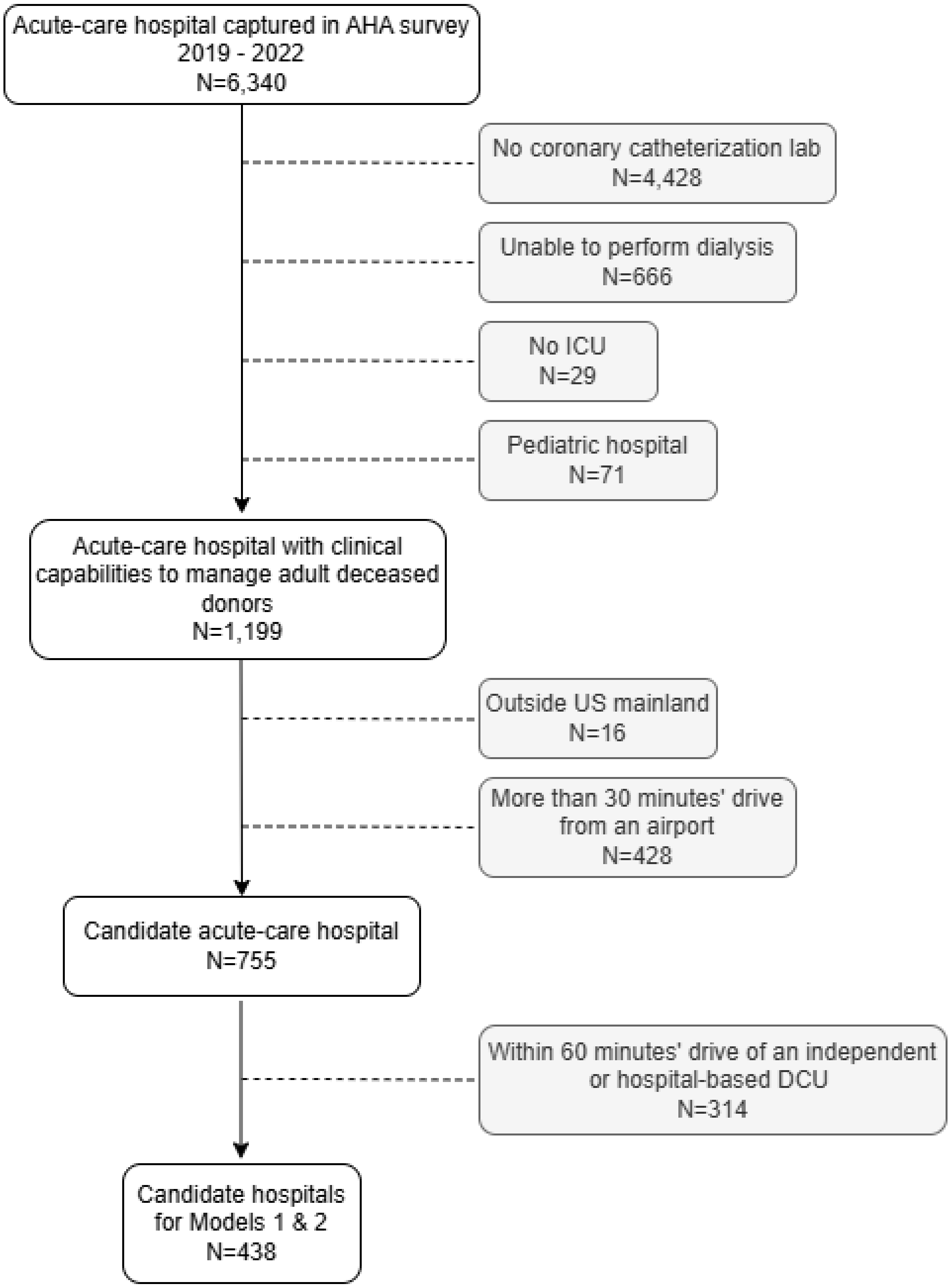

AHA: American Hospital Association, DCU: donor care unit, ICU: intensive care unit

© 2026 Potluri VS et al. *JAMA Network Open*.

eTable 1. Number of Candidate DCUs According to Maximum Driving Time From a DCU to an Airport

Maximum DCU to airport drive time	Candidate DCUs*
15 minutes	181
30 minutes	438
45 minutes	545
60 minutes	629

*All other acute-care hospital exclusion criteria were applied in the order depicted in the **eFigure 2**.

DCU: Donor Care Unit

© 2026 Potluri VS et al. *JAMA Network Open*.

eTable 2. Comparison of Transportation Efficiency and Geographic Coverage Following an Increase in Driving Time Boundaries

	Model 2: Ignoring OPO boundaries	Model 3: Ignoring OPO boundaries (increased airport drive time)	Model 4: Ignoring OPO boundaries (increased donor drive time)
**Maximum donor hospital – DCU drive time**	**3 hours**	**3 hours**	**4 hours**
**Maximum DCU – airport drive time**	**30 minutes**	**45 minutes**	**30 minutes**
**Existing DCUs**	34	34	34
**Proposed DCUs (primary outcome)**	22	22	17
**Secondary outcomes**			
**Total number of donors within the driving distance boundaries of a DCU (%)[b]**	51,213 (96.5%)	51,213 (96.5%)	51,756 (97.5%)
**Total number of donor hospital ZIP codes within the driving distance boundaries of a DCU (%)**	2,095 (95.1%)	2,095 (95.1%)	2,130 (96.7%)
**Median number of donors within the driving distance boundaries of each (existing and proposed) DCU (IQR)**	762 (430 – 1,138)	743 (430 – 1,138)	829 (492 - 1,353)
**Median number of donors covered by each projected DCU (IQR)**	475 (333 – 762)	475 (333 – 753)	499 (305 - 928)
**Median number of donor hospital ZIP codes within the driving distance boundaries of a DCU, (IQR)**	30 (19 - 46)	30 (19 - 46)	34 (21 - 57)
**Median driving time between donor hospitals and DCUs, weighted by number of donors in each hospital, minutes (IQR)**	50.5 (19.9 - 105.9)	51.8 (20 - 105.6)	59.4 (21.8 - 112.9)
**Median driving distance between donor hospitals and DCUs, weighted by number of donors in each hospital, miles (IQR)**	40.8 (11.3 - 105.2)	41 (11.6 - 104.9)	51.6 (13.4 - 110)

[a] As of December 31, 2023. Includes 12 independent and 22 hospital-based existing DCUs. When a donor was located within the 180-minute boundary of two DCUs in the same DSA, for distance calculations, we attributed them to the nearest DCU.

[b] Donor volume was calculated assuming all brain-dead donors within 180 minutes' drive are transferred over our study period (2018-2023)

DCU: donor care unit, DSA: donation service area, IQR: interquartile range, N/A: not applicable, OPO: organ procurement organization

© 2026 Potluri VS et al. *JAMA Network Open*.

eReferences

1. Vail EA, Tam VW, Sonnenberg EM, Lavu NR, Reese PP, Abt PL et al. Characterizing proximity and transfers of deceased organ donors to donor care units in the United States. *Am J Transplant* 2024;24(6):983-992.

2. Marklin GF, Brockmeier D, Spector K. The 20-year paradigm shift toward organ recovery centers: 2500 donors at Mid-America Transplant and broader adoption across the United States. *Am J Transplant.* Jul 2023;23(7):891-903. doi:10.1016/j.ajt.2023.01.010

© 2026 Potluri VS et al. *JAMA Network Open*.
