# Supplementary material for: Geographic Accessibility of Deceased Organ Donor Care Units
Source: JAMA Netw Open. 2026 Mar 13;9(3):e261703. doi: 10.1001/jamanetworkopen.2026.1703 (PMC12988448; doi:10.1001/jamanetworkopen.2026.1703)
Supplement: Supplement 2. — Data Sharing Statement [file jamanetwopen-e261703-s002.pdf]

Data Sharing Statement

Data
Data available: No

Additional Information
Explanation for why data not available: The data were collected at the individual patient level and are maintained by the Organ Procurement and Transplantation Network. Data are available upon request to the Organ Procurement and Transplantation Network at: https://optn.transplant.hrsa.gov/data/view-data-reports/request-data/
